# Phase behavior of ASDs based on hydroxypropyl cellulose

**DOI:** 10.1016/j.ijpx.2020.100070

**Published:** 2020-12-19

**Authors:** Christian Luebbert, Edmont Stoyanov, Gabriele Sadowski

**Affiliations:** a amofor GmbH, Otto-Hahn-Str. 15, D-44227 Dortmund, Germany; b Nisso Chemical Europe GmbH, Berliner Allee 42, D-40212 Düsseldorf, Germany; c TU Dortmund University, Laboratory of Thermodynamics, Emil-Figge-Str. 70, D-44227 Dortmund, Germany

**Keywords:** Amorphous solid dispersion, Solubility, Miscibility, PC-SAFT, Long-term stability, Hydroxypropyl cellulose

## Abstract

Novel polymeric carriers for amorphous solid dispersions (ASDs) are highly demanded in pharmaceutical industry to improve the bioavailability of poorly-soluble drug candidates. Besides established polymer candidates, hydroxypropyl celluloses (HPC) comes more and more into the focus of ASD production since they have the availability to stabilize drug molecules in aqueous media against crystallization. The thermodynamic long-term stability of HPC ASDs with itraconazole and fenofibrate was predicted in this work with PC-SAFT and compared to three-months enduring long-term stability studies. The glass-transition temperature is a crucial attribute of a polymer, but in case of HPC hardly detectable by differential scanning calorimetry. By investigating the glass transition of HPC blends with a miscible polymer, we were for the first time able to estimate the HPC glass transition. Although both, fenofibrate and itraconazole reveal a very low crystalline solubility in HPC regardless of the HPC molecular weight, we observed that low-molecular weight HPC grades such as HPC-UL prevent fenofibrate crystallization for a longer period than the higher molecular weight HPC grades. As predicted, the ASDs with higher drug load underwent amorphous phase separation according to the differential scanning calorimetry thermograms. This work thus showed that it is possible to predict critical drug loads above which amorphous phase separation and/or crystallization occurs in HPC ASDs.

## Graphical abstract

**Figure:**
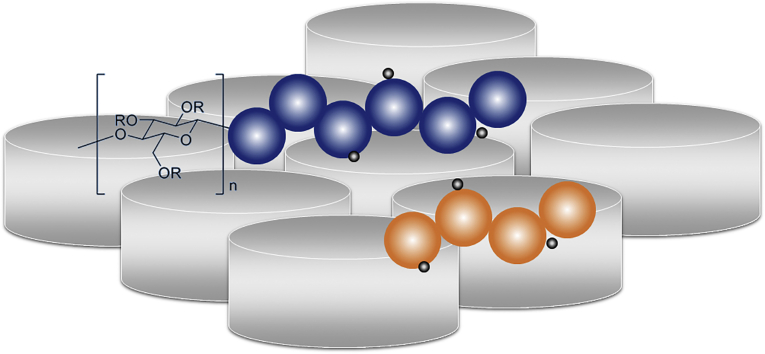

## Nomenclature

a: Helmholtz energy J mol^−1^
h: molar enthalpy J mol^−1^
c_p_: Heat capacity J (mol K)^−1^
M: molar mass g/mol
m: segment number -
k_B_: Boltzmann constant J K^−1^
k_i__j_: binary interaction parameter -
N_assoc_: number of association sites -
R: ideal gas constant J (mol K)^−1^
p: pressure bar
T: temperature K or °C
T_g_: glass-transition temperature K or °C
u: dispersion energy J
w_i_: mass fraction wt%
x_i_: mole fraction mol%
**Greek characters**
γ: activity coefficient -
ε^AiBi^: association energy J
ρ: density kg m^−3^
κ^AiBi^: association volume -
σ_seg_: segment diameter Å
**Subscripts**
i,j: component
int: intersection
**Superscripts**
assoc.: associating
disp: dispersion
hc: hard chain
L: liquid
res: residual
S: solid
V: vapor

## 1 Introduction

Amorphous solid dispersions (ASDs) play a major role as enabling formulations for poorly water-soluble new chemical entities (Vasconcelos et al., 2007; Chiou and Riegelman, 1971; Dhirendra et al., 2009). In such formulations, the active pharmaceutical ingredient (API) is molecularly dispersed in a polymer, that helps preventing the API from crystallization during long-term storage and facilitates the dissolution in aqueous media.

The polymer matrix shall on the one hand stabilize the amorphous form of the API and thus prevent API crystallization during storage, and on the other hand, it shall provide stabilizing dissolution properties in order to maintain high API concentrations in gastrointestinal fluids during dissolution (Konno et al., 2008; Anderson, 2018).

For formulators, it is of high interest to identify an optimal polymeric excipient for ASD formulations, e.g. by using completely new synthetic polymers (e.g. Soluplus®, a polyvinyl caprolactam-*co*-polyvinyl acetate-*co*-polyethylene glycol graft copolymer or the polymethacrylates and poly methacrylic acid based Eudragit® grades (Shamma and Basha, 2013)), or by utilizing polymer mixtures combining the advantages of different polymers (Monschke and Wagner, 2020; Lehmkemper et al., 2018a; Janssens et al., 2008).

Popular polymeric excipients like polyvinyl pyrrolidone (PVP) or the *co*polymer poly(vinylpyrrolidone-co-vinyl acetate) (PVPVA64) show high API solubilities and are thus theoretically able to stabilize the amorphous API during storage very well (Prudic et al., 2014a; Prudic et al., 2014b; Lehmkemper et al., 2017a; Tao et al., 2009; Sun et al., 2010). However, they are disadvantageous with respect to hydrophilicity (high amount of water absorbed at humid storage conditions leading to a destabilization and API crystallization (Qi et al., 2013; Saboo and Taylor, 2017; Chen et al., 2018a; Luebbert and Sadowski, 2017)) and their low potential to stabilize high API concentrations in aqueous media during dissolution (weak parachute effect) (Schittny et al., 2020). Cellulosic polymers like hydroxypropyl methylcellulose acetate succinate (HPMCAS) are popular in ASD applications since they better stabilize the API against recrystallization in aqueous media (Monschke and Wagner, 2020; Sun and Lee, 2015) (precipitation inhibitor). Unfortunately, API solubilities herein often are very low (Lehmkemper et al., 2017b; Tian et al., 2013) and thus those ASDs might crystallize within their shelf life. A promising polymer family fulfilling both stabilizing criteria might be hydroxypropyl celluloses (HPC), which were recently also considered as polymeric carriers in ASDs (Monschke and Wagner, 2020; Rashid et al., 2015; Sarode et al., 2014a). Despite the potential as growth inhibitor of crystal nuclei during storage and dissolution in aqueous media, HPC revealed higher ASD storage stabilities compared to PVPVA64 ASDs at lower storage temperature and elevated humidity (Sarode et al., 2014b).

In this work, we investigate the stabilization potential of HPC for application in ASDs. HPC is a modified cellulose already applied for several tablet-coating applications or as tablet binder (Picker-Freyer and Dürig, 2007) and recently also discussed as excipient for ASD applications (Sarode et al., 2014a; Osawa et al., 2014). It is soluble in many organic solvents and in water and thus applicable in spray-drying manufacturing processes.

The glass transition temperature (T_g_) and the API solubility in the polymer are two major aspects for achieving long-term stable ASDs (Anderson, 2018; Tian et al., 2013; Huang and Dai, 2014). A storage below the ASDs T_g_ enhances its kinetic stability via a reduced molecular mobility and thus API-crystallization velocity (Theil et al., 2017). An API load in the ASD above its solubility might cause crystallization (Tao et al., 2009; Tian et al., 2013), the occurrence of amorphous phase separation might lead to a local enrichment of amorphous API and accelerated crystallization (Saboo and Taylor, 2017; Marsac et al., 2010; Yang et al., 2013; Six et al., 2003; Luebbert et al., 2017). Both, crystallization and amorphous phase separation are highly unwanted as they alter the ASDs dissolution performance (Saboo et al., 2020; Purohit and Taylor, 2015; Tian et al., 2016; Chen et al., 2018b). PC-SAFT has shown in recent studies its strength in predicting amorphous phase separation in complex pharmaceutical systems (Luebbert et al., 2017; Dohrn et al., 2020; Dohrn et al., 2021). The impact of moisture on ASD stability (amorphous phase separation and crystallization) was successfully investigated recently (Luebbert and Sadowski, 2017; Luebbert et al., 2018a). Anderson gave a detailed review on the different existing computational methods and stressed that ‘ASDs are typically hydrogen-bonded systems’- the hydrogen bonds are explicitly considered by the association term within PC-SAFT (Anderson, 2018).

The T_g_ is an essential attribute of a polymer since it determines the mobility of molecules at given storage temperature conditions. However, only inconsistent information on the T_g_ of pure HPC is available, values between 19 °C (Rials and Glasser, 1988) and 124 °C (Sakellariou et al., 1985) are reported in literature. These differences can not only be explained by different molecular weight grades or analytical techniques, but instead show the difficulty in determining the Tg of HPC. For a more detailed review on the different glass transitions, the reader is referred to Nyamweya and Hoag (Nyamweya and Hoag, 2000).

The T_g_ of fast crystallizing APIs (Baird et al., 2010), organic solvents or even gases is often hard to determine due to spontaneous crystallization and a highly-unstable amorphous state. In such cases, the T_g_ may be estimated by mixing the compound in different ratios with a second compound and extrapolating The T_g_'s of mixtures with different compositions to the pure-component's T_g_ (Nyamweya and Hoag, 2000).

The long-term stability of an ASD with respect to crystallization and amorphous phase separation is predictable by thermodynamic phase diagrams. Numerous works have recently been published studying the phase behavior of ASDs with the polymers PVP, PVPVA64, Soluplus®, HPMC or HPMCAS, Eudragit® or poly (lactic-*co*-glycolic acids) (Lehmkemper et al., 2018a; Prudic et al., 2014a; Prudic et al., 2014b; Tao et al., 2009; Tian et al., 2013; Luebbert et al., 2017; Prudic et al., 2015; Lehmkemper et al., 2018b; Kapourani et al., 2019). However, to the best of our knowledge, no study evaluated so far the phase behavior of HPC ASDs. Therefore, we investigate in this work the potential of different HPC grades to stabilize the amorphous state of APIs by thermodynamic modelling using the Perturbed-Chain Statistical Associating Fluid Theory (PC-SAFT). The predicted phase diagrams were validated via twelve-weeks-enduring long-term studies of spray-dried ASDs, during which the crystallization was monitored weekly.

## 2 Materials and Methods

### 2.1 Materials

Four different molecular weights (grades) of the polymer HPC (HPC-UL with 20,000 g/mol, HPC-SSL with 40,000 g/mol, HPC-SL with 100,000 g/mol, and HPC-L with 140,000 g/mol) were provided by Nisso Chemical Europe GmbH (Düsseldorf, Germany). The APIs fenofibrate (98% purity) and itraconazole (99% purity) were obtained from VWR International GmbH (Darmstadt). The solvents for DVS analysis (ethanol, acetone, and cyclohexane) were obtained in chromatographic grade from VWR International GmbH (Darmstadt), PVPVA64 was provided by BASF SE (Ludwigshafen, Germany). Water required for sorption experiments was filtered and deionized prior use.

### 2.2 Experimental Methods for characterizing the thermodynamic properties of pure HPC

The so-far unknown T_g_ of pure HPC was assessed indirectly since no glass-transition temperature is observable in differential scanning calorimetry (DSC) measurements of pure HPC. According to the supplier of HPC, the polymer PVPVA64 is fully miscible with HPC and shows in a DSC thermogram a clearly distinguishable T_g_ of 109 °C. PVPVA64 and HPC were spray dried in ratios of 10 wt%, 25 wt%, 50 wt%, 75 wt%, and 90 wt% (Büchi B290 spray dryer, Flawil, Switzerland). The inlet temperature of the spray dryer was set to 80 °C, the feed rate of the spray-dried solution was set to 7 ml/min and nitrogen was fed with a volume flow of 550 l/h to the atomizer nozzle. In total, 500 mg of solid (PVPVA64 and HPC) were weighted with an accuracy of ±0.1 mg in the desired PVPVA64/HPC ratio, dissolved in 50 ml ethanol and spray dried. A secondary drying was conducted afterwards for two days under vacuum conditions.

The T_g_ of pure HPC was then estimated by extrapolating the T_g_'s of the mixtures with the Gordon-Taylor-Eq. (Gordon and Taylor, 1952) to that of pure HPC. All DSC measurements were performed with a Q2000 (TA Instruments, Newcastle, USA) temperature-calibrated with pure indium. The DSC cell was purged with a stream of 50 ml/min nitrogen. A heat (2 K/min) -cool (10 K/min) -heat (2 K/min) procedure was performed for each sample, heating was carried out in modulated heating-only mode (oscillation amplitude 0.318 K, oscillation period 60 s) and the glass transition was determined in the second heating ramp to ensure that eventually remaining residual solvent or moisture was removed. The DSC thermograms were evaluated with the Software TA Universal Analysis (TA Instruments, Newcastle, USA).

The sorption of solvents with different polarity in HPC-UL was measured to determine PC-SAFT pure-component parameters for HPC-UL (Lehmkemper et al., 2017b; Reschke et al., 2014). The solvents acetone, ethanol, and cyclohexane were selected as solvents for the dynamic vapor sorption (DVS) analysis. HPC-UL samples were exposed to 15%, 30%, 45%, 60%, 75% and 90% relative saturation (RS) (RS = *p*_*i*_/*p*_*oi*_^*LV*^). RS was adjusted by mass-flow controllers mixing a stream of saturated vapor with dry nitrogen in the desired ratios. Prior to analysis, all samples were dried with dry nitrogen to remove residuals from the samples and the sample dry mass was determined. The equilibrium condition of the DVS device was set to a relative mass change of dm/dt < 0.0001%/min, the minimum stage time was 120 min. The total gas flow rate was set to 100 cm^3^/min.

Since the melt densities of pure HPC were not available and are not easy to determine, we fitted the PC-SAFT parameters of HPC-UL to the density of HPC-UL/water mixtures. Densities of HPC-UL/water mixtures with different HPC-UL concentrations (10 wt% and 20 wt%) were determined at 15 °C, 25 °C, 35 °C, and 45 °C with an Anton Paar Densimeter DMA 4100 (Graz, Austria). The uncertainty of the density measurement was 0.0001 g/cm^3^ at a temperature uncertainty of 0.03 K.

### 2.3 Experimental Methods for determining the phase diagrams of API/HPC ASDs

The solubility of APIs in HPC was determined via heat-cool-heat DSC measurements of ball-milled API/HPC mixtures with different API loads. Solubility temperatures were determined as the dissolution endpoint of the first heating ramps, which is considered as the temperature at which all API crystals dissolved in the polymer. These solubility temperatures were determined at 1 K/min, 2 K/min and 5 K/min heating rates from the heat flow signals and linearly extrapolated to the equilibrium value at 0 K/min (Tao et al., 2009). The T_g_'s of the mixtures were determined afterwards in the second heating ramp from the reversing heat flow signal at 2 K/min.

### 2.4 Experimental methods for preparing ASDs and characterizing ASDs during long-term studies

ASDs with 5 wt%, 15 wt% and 30 wt% API content were manufactured by spray drying (Büchi B290, Flawil, Switzerland). Inlet temperature, feed rate of the peristaltic pump and nitrogen stream were the same parameters as described in Section 2.2. In total, 1 g of solid (API and HPC) were weighted with an accuracy of ±0.1 mg in the desired API/HPC ratio, dissolved in 100 ml ethanol and spray dried.

All spray-dried ASDs were subjected to long-term stability studies for twelve weeks at 25 °C and 0% relative humidity (in vacuum chambers). Each ASD was weekly analyzed for the occurrence of crystals via powder X-ray diffraction (PXRD) and DSC. The minimum XRD detection limit of fenofibrate crystals was 0.4 wt%.

DSC and XRD are regarded as complementary when detecting the crystallinity in ASDs and only a combination of both methods yields a robust information on the actual degree of crystallinity. XRD has a high detection limit of crystals (e.g. Greco et al. reports a detection limit of 3% crystals (Greco et al., 2012)), and lower amounts of crystals can hardly be detected by that measurement technique. On the other hand, DSC can detect smallest traces of crystallinity but destroys the sample during heating it up (thermal degradation or recrystallization upon heating). We therefore decided to utilize both methods complementary. Crystallinity in the ASDs was additionally quantified via DSC by a linear heating ramp of 10 K/min from room temperature to 20 K above the melting temperature of the respective API. The measured melting enthalpy ($\Delta h_{\mathrm{ASD}}^{\mathrm{SL}}$) divided by the product of pure-APIs melting enthalpy ($\Delta h_{\mathrm{API}}^{\mathrm{SL}}$, Table 5) and API mass fraction in the ASD (w_API_) yielded the crystallinity in the ASD (*crystallinity* = $\Delta h_{\mathrm{ASD}}^{\mathrm{SL}}$/($\Delta h_{\mathrm{API}}^{\mathrm{SL}}$ · *w*_*API*_).

The PXRD measurements were carried out with approximately 5 mg of each ASD poured on a silicon sample holder in a Rigaku MiniFlex 600 PXRD (Tokyo, Japan). Samples were scanned in a range of 5° < 2Ɵ < 30°.

### 2.5 Phase diagram modelling with PC-SAFT

The crystalline solubility of an API in HPC is calculated with Eq. 1 (Prausnitz et al., 1999).

(1) $$x_{\mathrm{API}}=\frac{1}{\gamma_{\mathrm{API}}}\cdot \exp\left[-\frac{\Delta h_{\mathrm{API}}^{\mathrm{SL}}}{R\cdot T}\cdot \left(1-\frac{T}{T_{\mathrm{API}}^{\mathrm{SL}}}\right)-\frac{\Delta c_{p,\mathrm{API}}^{\mathrm{SL}}}{R}\left[\ln\left(\frac{T_{\mathrm{API}}^{\mathrm{SL}}}{T}\;\right)-\frac{T_{\mathrm{API}}^{\mathrm{SL}}}{T}+1\right]\right]$$

Here, *x*_*API*_ is the mole-fraction solubility of the API in the liquid phase. The activity coefficient of the API *γ*_*API*_ accounts for all intermolecular interactions between the API and HPC and was obtained in this work from PC-SAFT. The melting properties of the solute are the melting temperature (*T*_*API*_^*SL*^), the melting enthalpy ($\Delta h_{\mathrm{API}}^{\mathrm{SL}}$), and the difference of the heat capacities of the solid and liquid API (*Δc*_*p*, *API*_^*SL*^). R is the ideal gas constant (8.3145 J (mol K)^−1^).

Amorphous phase separation (separation of a mixture into two liquid phases L1 and L2) is calculated by Eq. 2.

(2) $$x_{i}^{L1}\cdot \gamma_{i}^{L1}=x_{i}^{L2}\cdot \gamma_{i}^{L2}\;$$

This equation was solved simultaneously for each component i in the mixture (e.g. for API as well as for HPC).

The activity coefficients required for phase diagram modelling were obtained in this work using PC-SAFT. PC-SAFT is a thermodynamic model which treats molecules as chains of spherical segments. Each molecule has a defined number of segments (segment number *m*^*seg*^) with segment diameter *σ* and a dispersion energy parameter *u*/*k*_*B*_ describing the segment-segment interaction. PC-SAFT calculates the residual Helmholtz energy *a*^*res*^ by summing up different contributions caused by repulsion (hard chain *a*^*hc*^), attraction (dispersion *a*^*disp*^) and association (*a*^*assoc*^) of the molecules (Eq. 3). The detailed expressions of the contributions can be found in literature (Gross and Sadowski, 2002; Tumakaka et al., 2002; Gross and Sadowski, 2001).

(3) $$a^{\mathrm{res}}=a^{\mathrm{hc}}+a^{\mathrm{disp}}+a^{\mathrm{assoc}}$$

Contributions from interactions between unlike molecule species i and j in a mixture are calculated via the Berthelot-Lorentz mixing rules given in Eqs. (4), (5).

(4) $$\sigma_{\mathrm{ij}}=\frac{1}{2}\;\left(\sigma_{i}+\sigma_{j}\right)$$

(5) $$u_{\mathrm{ij}}=\left(1-k_{\mathrm{ij}}\right)\sqrt{u_{i}u_{j}}$$

The dispersion energy u_ij_ is corrected via the interaction parameter *k*_*ij*_ which is fitted to experimental binary data. *k*_*ij*_ might be a constant value or linearly depends on temperature as expressed in Eq. 6.

(6) $$k_{\mathrm{ij}}=k_{\mathrm{ij},\mathrm{int}}+k_{\mathrm{ij},\mathrm{slope}}\cdot T\;\left[K\right]$$

Hydrogen bonds formed between molecules like water or APIs are considered via a defined number of donor/acceptor sites *N*^*assoc*^. Accounting for hydrogen-bond formation between these sites requires two more model parameters, namely the association energy *ε*^*AB*^/*k*_*B*_ and the association volume *κ*^*AB*^. Cross association in mixtures of associating components was considered by applying mixing rules presented in Eqs.7 and 8.

(7) $$\varepsilon^{A_{i}B_{j}}=\frac{1}{2}\;\left(\varepsilon^{A_{i}B_{i}}+\varepsilon^{A_{j}B_{j}}\right)$$

(8) $$\kappa^{A_{i}B_{j}}=\sqrt{\kappa^{A_{i}B_{i}}\kappa^{A_{j}B_{j}}}{\left[\frac{2\sigma_{\mathrm{ii}}\sigma_{\mathrm{jj}}}{\left(\sigma_{\mathrm{ii}}+\sigma_{\mathrm{jj}}\right)}\right]}^{3}$$

PVPVA64 does not self-associate with molecules of the own kind but nevertheless act as proton donor and acceptor when mixed with a self-associating component. For the molecule fenofibrate, *ε*^*AB*^ was set to zero and *κ*^*AB*^ was set to the value 0.02 (Brinkmann et al., 2019).

The T_g_ of the ASDs as function of API mass fraction w_API_ was modelled using the Gordon-Taylor Equation (Eq. 9) (Gordon and Taylor, 1952):

(9) $$T_{g}=\frac{w_{\mathrm{API}}\;T_{g,\mathrm{API}}+K_{\mathrm{GT}}w_{\mathrm{HPC}}\;T_{g,\mathrm{HPC}}}{w_{\mathrm{API}}+K_{\mathrm{GT}}\;w_{\mathrm{HPC}}}$$

T_g_ follows the Gordon-Taylor equation only in miscible mixtures, thus T_g_ is only modelled in those regions. For the PVPVA64/HPC-blends, the API in Eq. 9 is replaced by PVPVA64. The binary Gordon-Taylor parameter K_GT_ was either fitted to the obtained DSC data (in case of the PVPVA64/HPC blends) or (in case of the ASDs) predicted using the correlation *K*_*GT*_ = *ρ*_*API*_*T*_*g*__*,*__*API*_/*ρ*_*HPC*_*T*_*g*, *HPC*_ (*ρ* is the density of the amorphous substances).

## 3 Results

### 3.1 Glass-transition temperatures of pure HPC grades

As mentioned in the introduction, the T_g_ of pure HPC is hardly detectable since the step height of the heat capacity at the glass transition is nearly zero (a typical DSC thermogram of a HPC-UL sample is discussed in the supplement in Fig. S1).

**Fig. 1:**
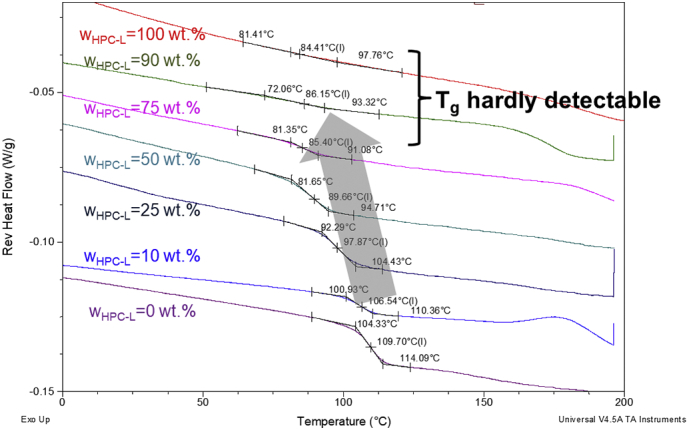
DSC thermograms (reversing heat flow) of HPC-L/PVPVA64-blends.

The glass-transition temperatures of spray-dried HPC/PVPVA64 blends with 10 wt%, 25 wt%, 50 wt%, 75 wt% and 90 wt% HPC were investigated in this work (blends were prepared for all HPC grades). The measured DSC thermograms of the HPC/PVPVA64 blends are summarized in Fig. 1 (second heating ramp of a heat-cool heat procedure, compare Section 2.2). The measured glass-transition temperatures decrease from 109.7 °C in pure PVPVA64 upon addition of HPC-L to 84.41 °C in pure HPC-L (a broad glass-transition was detected by the software TA Universal Analysis, though it is not observable with the eye). The step heights at the T_g_ decrease with increasing HPC content. The T_g_ of pure HPC-L and the sample with 90 wt% HPC-L is not detectable anymore with the eye, whereas the T_g_'s in blends with up to 75% HPC-L can be well detected visually.

**Fig. 2:**
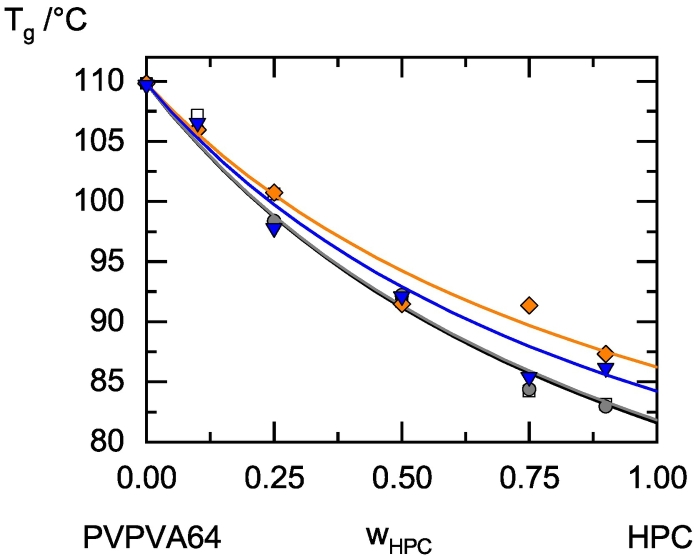
Measured T_g_ as function of HPC content (symbols) and glass-transition modelled via Gordon-Taylor equation (lines). Squares indicate blends with HPC-UL, circles indicate blends with HPC-SSL, diamonds indicate blends with HPC-SL and triangles indicate blends with HPC-L.

The glass-transition temperature obtained from those measurements is shown in Fig. 2 for all HPC/PVPVA64 blends as function of composition.

By fitting the Gordon-Taylor parameter and extrapolating to the unknown T_g_ of the pure HPC grades, we were able to estimate the T_g_'s of all four investigated HPC grades, they are shown in Table 1. The HPC T_g_'s increase slightly with increasing HPC molecular weight (except for HPC-L). The extrapolated Tg's still are slightly error-prone due to the limitations of the Gordon-Taylor-approach in correctly describing the concentration-dependency of the Tg in a polymer blend. However, also modelling approaches like that of Brostow et al. did not improve the overall modelling acuracy (Brostow et al., 2008).

**Table 1:** Glass-transition temperatures of HPC grades determined via extrapolating the glass transition of spray dried HPC/PVPVA64 blends.

HPC grade	M_W_/g/mol	T_g_/°C
UL	20,000	81.6
SSL	40,000	81.8
SL	100,000	86.2
L	140,000	84.2

The T_g_ values reported in literature (19 °C (Rials and Glasser, 1988) – 124 °C (Dave et al., 1995)) strongly differ from our extrapolated values. Nyamwega and Hoang (Nyamweya and Hoag, 2000) in detail discussed possible reasons for the discrepancies among the literature values (e.g. different experimental techniques such as dynamic-mechanical analysis, torsional braid analysis or DSC, different polymer suppliers, different molecular weights, enthalpy relaxation, sample preparation etc.). The same authors tried to assess the T_g_ of HPC via an extrapolation with blends of HPMC E5 and HPC. Unfortunately, they were not successful as they could not observe any change of T_g_ upon addition of HPC. They assumed that the chosen polymer HPMC E5 is unexpectedly immiscible with HPC and thus not appropriate for an extrapolation. Karari et al. also did not succeed with a dynamic-mechanical determination of the Tg of HPC (Kararli et al., 1990). Selecting PVPVA64 as blend component in this work, we were now able to estimate T_g_'s of the HPC grades.

### 3.2 PC-SAFT pure-component parameter determination of HPC grades

The PC-SAFT pure-component parameters of HPC-UL were fitted simultaneously to sorption data in organic solvents with different polarity and to densities of ethanol/HPC-UL mixtures.

#### 3.2.1 Sorption of organic solvents in HPC-UL

**Fig. 3:**
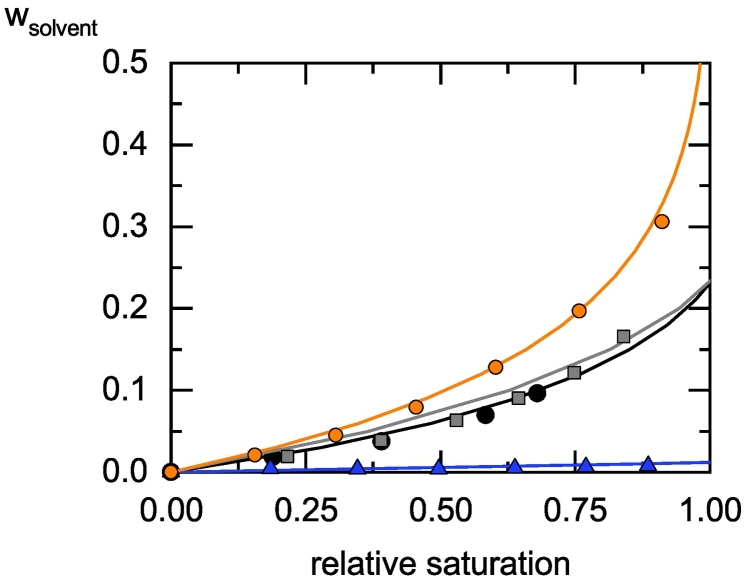
Equilibrium solvent sorption in HPC-UL at a temperature of 25 °C. Symbols are DVS-determined experimental values, lines are modelling results with PC-SAFT. Ethanol sorption data is indicated by circles, acetone sorption data by squares, water sorption data by black squares and cyclohexane data by triangles.

The equilibrium vapor sorption of the solvents as function of RS is summarized in Fig. 3.

It can be seen that HPC-UL absorbs most ethanol (e.g. w_ethanol_ = 0.1972 absorbed at 0.75 RS), followed by a lower absorption affinity of acetone and water and extremely low absorption of cyclohexane (e.g. w_cyclohexane_ = 0.0061 at 0.77 RS). The equilibrium sorption data of water and acetone are almost identical. HPC-UL absorbs almost no cyclohexane. The exact values of the measured absorption equilibria are given in the supplement Table S1.

#### 3.2.2 Density of HPC-UL/water mixtures

Density values of the pure compound are essential for estimating the geometric parameters of the PC-SAFT modelling. Since the melt densities of the pure polymers are often not available, PC-SAFT parameters were alternatively fitted to the density of HPC-UL/solvent mixtures. The results are summarized in Table 2.

**Table 2:** Measured densities of HPC-UL/water mixtures at different temperatures.

w_HPC-UL_	Density/g cm^−3^	Temperature /°C
0.0	0.9994	15
0.1	1.0206	15
0.2	1.0444	15
0.0	0.9973	25
0.1	1.0176	25
0.2	1.0411	25
0.0	0.9943	35
0.1	1.0150	35
0.2	1.0369	35
0.0	0.9909	45
0.1	1.0106	45
0.2	1.0323	45

The density linearly increases with increasing HPC-UL mass fraction in the solution and decreases with increasing temperature.

#### 3.2.3 PC-SAFT parameters

**Fig. 4:**
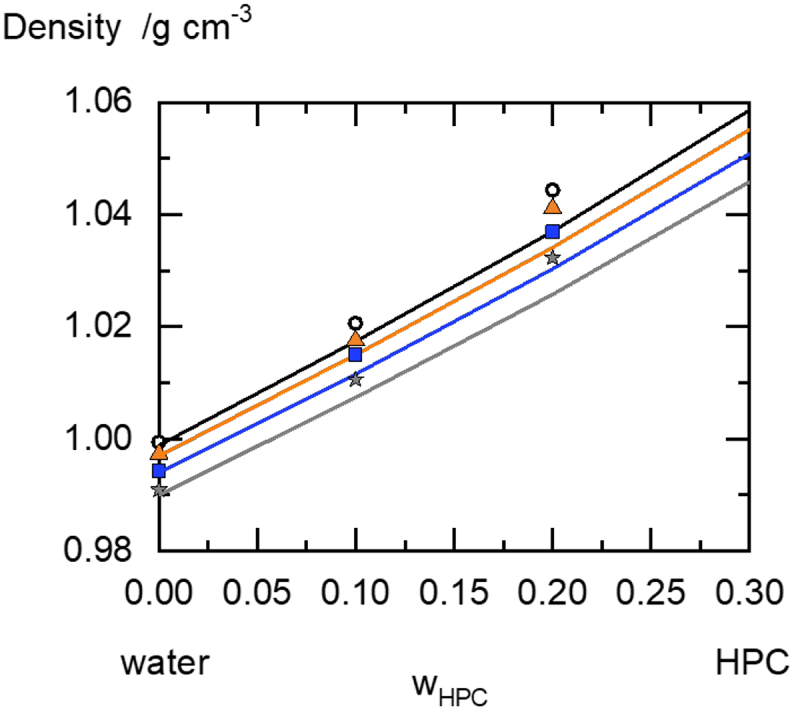
Density of HPC-UL/mixtures at different temperatures. Stars correspond to 15 °C, squares correspond to 25 °C, triangles correspond to 35 °C and circles correspond to 45 °C. The experiments are marked by symbols; the lines are the PC-SAFT calculations.

The PC-SAFT pure-component parameters of HPC-UL were fitted to vapor-sorption data (Fig. 3) and densities of HPC-UL/water mixtures (Fig. 4) and are shown in Table 3. Parameters of the APIs fenofibrate and itraconazole were obtained from literature.

**Table 3:** PC-SAFT pure-component parameters of HPC-UL, fenofibrate, and itraconazole.

Substance	m_i_^seg^/M_W_ /mol g^−1^	σ_i_ /Å	u_i_/k_B_/K	ε^AiBi^/k_B_ /K	κ^AiBi^	N_i_^assoc^ (donors/acceptors)	Parameter Ref.
HPC-UL	0.0446738	2.974	205.0	1600.0	0.02	286/286	This work
fenofibrate	0.0106957	4.767	244.8	0	0.02	0/2	(Brinkmann et al., 2019)
itraconazole	0.037	2.166	252.346	1204.88	0.02	2/2	(Paus et al., 2015)

PC-SAFT pure-component parameters of HPC grades with different molecular weight were obtained by altering the number of segments in the polymer chain. HPC-UL (20,000 g/mol) e.g. has a segment number of *m*_*HPC*−*UL*_^*seg*^ = 893.48, HPC-SSL (40,000 g/mol) has a segment number of *m*_*HPC*−*SSL*_^*seg*^ = 1786.95, HPC-SL (100,000 g/mol) has a segment number of *m*_*HPC*−*SL*_^*seg*^ = 4467.38 and HPC-L (140,000 g/mol) has a segment number of *m*_*HPC*−*L*_^*seg*^ = 6254.33. All other PC-SAFT parameters remain constant and do not change with changing molecular weight. The via PC-SAFT calculated density of pure HPC-UL at 25 °C is 1.216 g/cm^3^, this lies in a reasonable range for a polymer and thus further validates the fitted parameters.

The values for the PC-SAFT binary interaction parameters k_ij_ are summarized in Table 4. These values are also valid for all HPC grades.

**Table 4:** Binary PC-SAFT interaction parameters (k_ij_) between HPC and the other investigated components.

Water	Cyclohexane	Acetone	Ethanol	Fenofibrate	Itraconazole
−0.0623	0.0680	−0.0050	0	0	−0.037

The melting properties required for calculating the crystalline-API solubilities (Eq. 1) were obtained from literature (Table 5).

**Table 5:** Melting properties and T_g_'s of the APIs fenofibrate and itraconazole obtained from literature.

	Fenofibrate	Itraconazole
$\Delta h_{\mathrm{API}}^{\mathrm{SL}}$ /J/g	92.93 (Watterson et al., 2014)	98.78 (Paus et al., 2015)
*T*_*API*_^*SL*^ /°C	80.78 (Brinkmann et al., 2019)	168.75 (Paus et al., 2015)
T_g,API_ /°C	−18.44 (this work)	58.16 (Paus et al., 2015)
*Δc*_*p*, *API*_^*SL*^ /J (mol K)^−1^	124.3(Watterson et al., 2014)	177.8 (Paus et al., 2015)

### 3.3 Phase behavior of HPC-containing ASDs

The phase diagram of the fenofibrate/HPC-UL ASD was modelled with PC-SAFT using the PC-SAFT parameters summarized in Table 3 and the APIs melting properties presented in Table 5.

**Fig. 5:**
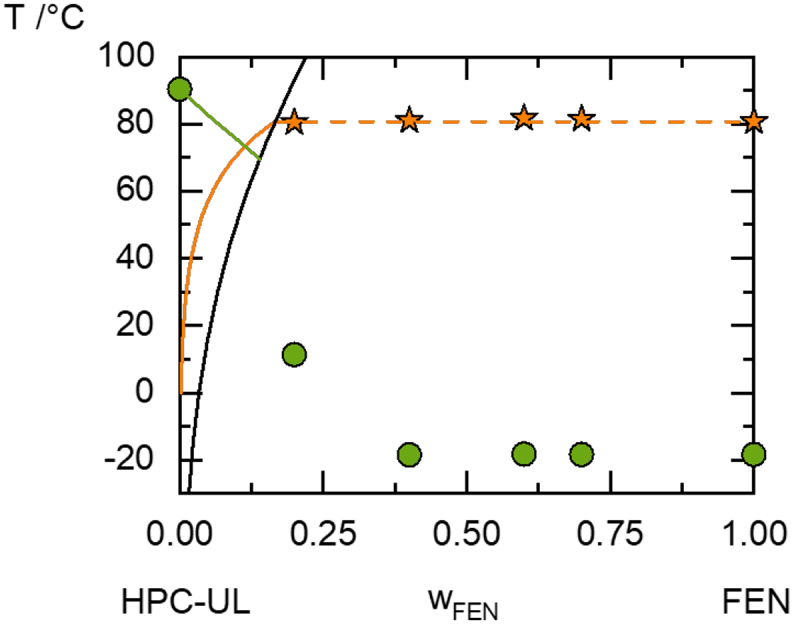
Phase diagram of fenofibrate/HPC-UL measured via DSC (symbols) and predicted with PC-SAFT (lines). The orange line is the solubility of fenofibrate in HPC-UL, the dashed orange line is a metastable solubility line, the black line is the amorphous solubility of fenofibrate in HPC-UL and the green line is the T_g_ of the amorphous ASDs. The circles are experimentally-determined T_g_'s and the stars are solubility temperatures measured via DSC. (For interpretation of the references to colour in this figure legend, the reader is referred to the web version of this article.)

The PC-SAFT calculation and all DSC measurements are summarized in the phase diagram shown in Fig. 5.

As can be seen, the measured solubility temperatures did not change for different fenofibrate loads in the ASDs compared to the melting temperature of pure fenofibrate (80.78 °C). The reason for this is an amorphous phase separation region (right of the PC-SAFT predicted black line in Fig. 3)- all ASDs within this temperature/composition range will undergo amorphous phase separation. Indeed, the experimentally-determined glass transitions of ASDs with fenofibrate mass fractions above 0.4 showed the same value as that for pure fenofibrate. This is a hint that those ASDs undergo amorphous phase separation and almost pure fenofibrate precipitates amorphously from the mixture with HPC-UL. Thus, the phase-diagram prediction (k_ij_ = 0) very well agrees with the observed phase behavior. Tg only follows Gordon-Taylor behavior in miscible blends, thus it was only calculated outside the region of amorphous phase separation.

As can be seen, the phase diagram can be predicted very well without fitting a binary interaction parameter (k_ij_ = 0). The predicted phase diagram reveals that the crystalline fenofibrate equilibrium solubility in HPC-UL at room temperature is 0.8 wt% and that thus all ASDs with higher fenofibrate loads are expected to crystallize during storage. Additionally, fenofibrate ASDs with fenofibrate loads above 5.8 wt% will undergo amorphous phase separation. This phenomenon was also visually observed in the DSC samples, where low-viscous fenofibrate covered the bottom of the sample pans and optically segregated from the polymer matrix. The glass transition temperature of the ASDs was predicted for the composition/temperature range in which no phase separation occurs (left side of the diagram). A comparable phase behavior is known from ASDs of ibuprofen and PLGA (Luebbert et al., 2017).

**Fig. 6:**
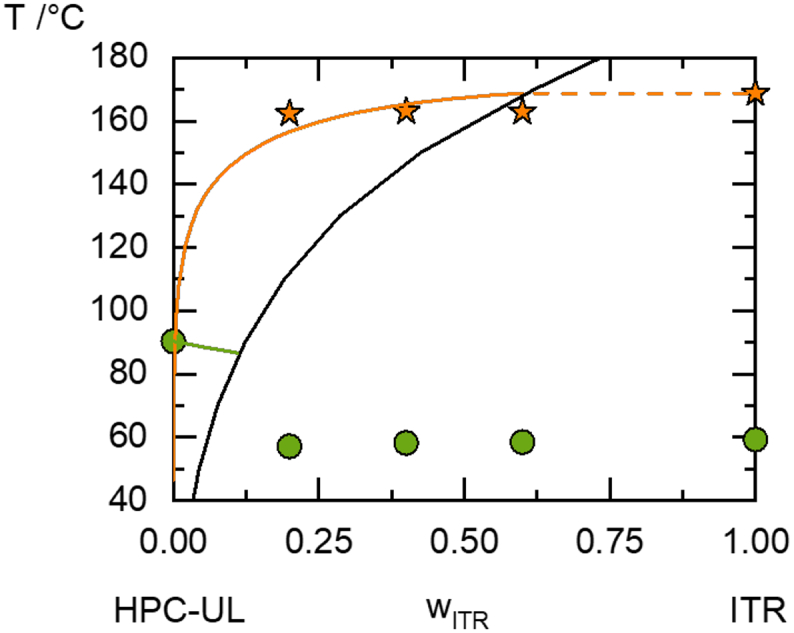
Phase diagram of itraconazole/HPC-UL measured via DSC and modelled with PC-SAFT. The orange line is the PC-SAFT calculated solubility of itraconazole in HPC-UL, the dashed orange line is the metastable solubility, the black line is the PC-SAFT predicted amorphous solubility of itraconazole in HPC-UL and the green line is the predicted T_g_ of the amorphous ASDs. Circles are T_g_'s determined via DSC; stars are solubility temperatures determined via DSC. (For interpretation of the references to colour in this figure legend, the reader is referred to the web version of this article.)

The PC-SAFT calculation and the DSC measurements of itraconazole/HPC-UL ASDs are summarized in the phase diagram shown in Fig. 6. The binary interaction parameter k_ij_ for this system was fitted to −0.037. The solubility temperatures of the ASDs slightly decrease relative to the melting temperature of pure itraconazole (168.75 °C). According to the PC-SAFT calculation, the extrapolated solubility of itraconazole in HPC-UL at 25 °C is only 0.001669 wt%.

All investigated ASDs revealed a glass-transition temperature close to that of pure itraconazole. This is no direct evidence for amorphous phase separation as for example Raman Imaging (Luebbert et al., 2018b), but a strong indirect hint that also itraconazole is not fully miscible with HPC-UL and at least some of the here-investigated ASDs underwent amorphous phase separation (Nyamweya and Hoag, 2000; Brostow et al., 2008; Kim et al., 2003).

Using the kij value fitted to the solubility data in Fig. 6, an amorphous phase separation was predicted. The predicted amorphous-phase-separation region agrees very well with the behavior observed in the DSC: In agreement with the DSC measurements, the PC-SAFT modelling predicts almost no melting point depression for all itraconazole concentrations in the ASD. The low solubility of itraconazole in HPC-UL (w_itraconazole_ = 0.001669 wt%) reveals that ASDs with higher itraconazole loads might crystallize during storage (at least after infinite time). Additionally, PC-SAFT predicts that ASDs with itraconazole loads above 2.0 wt% will undergo amorphous phase separation. According to the PC-SAFT predictions, the itraconazole-rich phase contains almost pure itraconazole. As the itraconazole-rich phase does contain almost no polymer that could prevent spontaneous nucleation, it can be expected that this phase crystallizes as quickly as pure amorphous itraconazole.

### 3.4 Influence of HPC molecular weight on the amorphous and crystalline API solubility

**Fig. 7:**
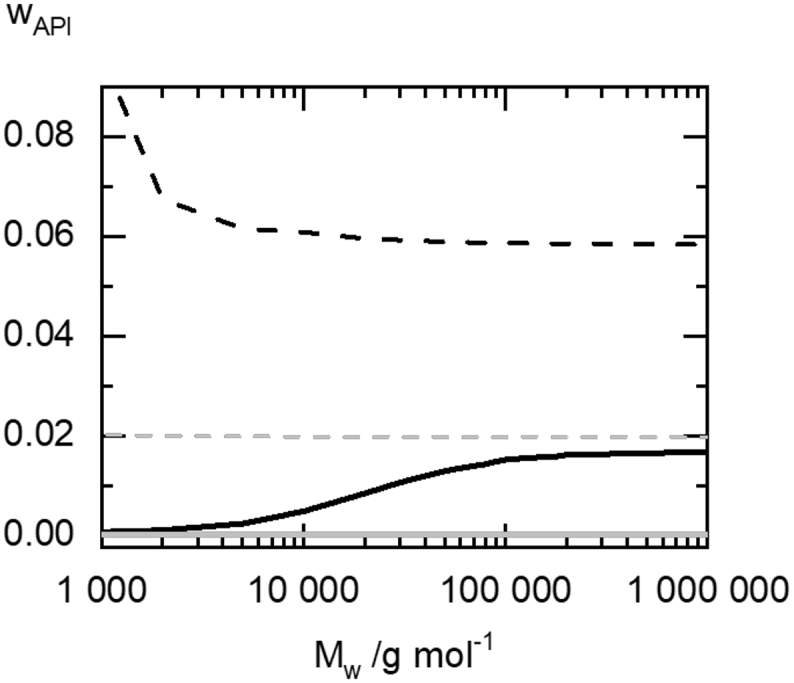
Solubility of fenofibrate (black lines) and itraconazole (gray lines) in HPC as function of HPC molecular weight at 25 °C. The crystalline solubilities are indicated as solid lines, the amorphous solubilities are indicated as dashed lines.

The influence of HPC molecular weight on the crystalline and amorphous solubility of fenofibrate and itraconazole in HPC was predicted using PC-SAFT in a range from 1000 g/mol to 1000,000 g/mol. The amorphous solubility is the concentration of API in the polymer above which amorphous phase separation occurs. It is predicted with Eq. 2 using the same PC-SAFT parameters as for calculations of crystalline solubility). Fig. 7 shows the result of this calculation at a temperature of 25 °C.

According to the prediction, the solubility of crystalline-fenofibrate increases sigmoidally on a log(M_w_)-scale from 0.07 wt% at a molecular weight of 1,000 g/mol to 1.68 wt% at 1000,000 g/mol. At the same time, the solubility of the amorphous API (i.e. the API concentration in the HPC-rich phase of a demixed ASD; compare Fig. 5) decreases. The investigated HPC grades of 20,000 g/mol – 140,000 g/mol are found in the middle of the calculated Mw range. Thus, amorphous phase separation will not occur in HPC grades with low molecular weight and the tendency of the ASD to undergo amorphous phase separation increases with increasing molecular weight of HPC.

According to the PC-SAFT predictions, the molecular-weight influence on the solubilities of crystalline and amorphous itraconazole and fenofibrate is quite different: The predicted crystalline itraconazole solubility remains constantly low at w_i__traconazole_ = 0.001669 wt% and is not affected by HPC molecular weights in the range from 1,000 g/mol to 1000,000 g/mol. The same holds true for the solubility of amorphous itraconazole which is not affected by HPC molecular weight and remains about 2 wt%.

### 3.5 Long-term stability of fenofibrate/HPC and itraconazole/HPC ASDs

ASDs with API loads of 5 wt%, 15 wt%, and 30 wt% were prepared for each API/HPC combination. The spray-dried ASDs were stored at 25 °C/0% RH and weekly analyzed via DSC and XRD for crystallinity. According to the PC-SAFT predictions (Fig. 5 and Fig. 6), all prepared ASDs were supersaturated and thus were expected to crystallize during storage.

**Fig. 8:**
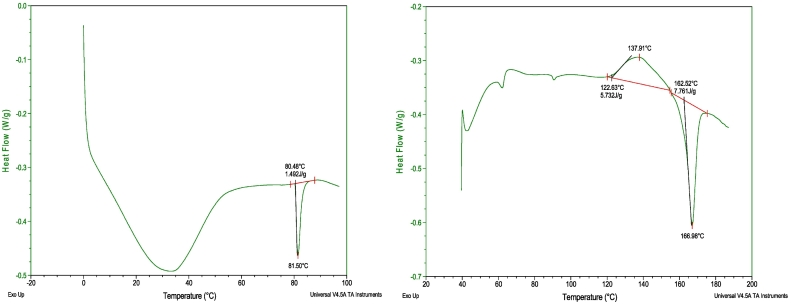
Example DSC thermograms of (a) a fenofibrate/HPC-UL ASD with 15 wt% fenofibrate after twelve weeks of storage and (b) of an itraconazole/HPC-UL ASD with 30 wt% itraconazole after 10 weeks of storage.

The evaluation of a DSC heat-flow signal of an HPC-UL/fenofibrate 15% ASD after 12 weeks of storage is shown as an example in Fig. 8. The melting enthalpy determined from this measurement was 1.492 J/g, which corresponds to a crystallinity of 1.492 / (92.93 ∙ 0.15) = 10.7%.

The melting enthalpies of itraconazole/HPC-ASDs were often hard to quantify since recrystallization occurred during heating. In those cases, the recrystallization enthalpy was subtracted from the melting enthalpy (Fig. 8b). In this example, the crystallinity was (7.761–5.732)/(98.78∙ 0.30) = 19.3% according to the DSC measurement while the corresponding PXRD measurement did not reveal any crystals. The recrystallization occurring during heating made a reliable interpretation of the DSC baseline impossible and lead to error-prone crystallinity values for several itraconazole/HPC-ASDs (bold values in Table 6). In case that recrystallization occurred during the DSC measurements, we considered the PXRD measurements as being the more reliable ones. In case that no recrystallization occurred during the measurements, the DSC measurements are certainly more accurate and even allowed quantifying smallest amounts of crystallinity (FEN/HPC ASDs).

**Table 6:**
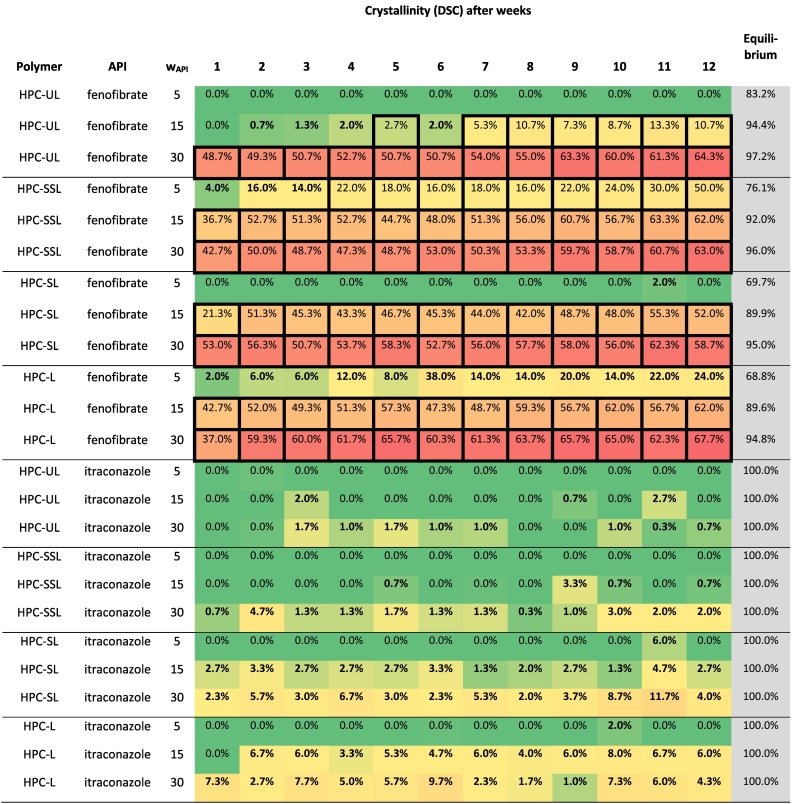
Long-term storage results of the API/HPC ASDs. Numbers indicate the crystallinity determined via DSC. X-ray crystalline samples are marked by bordered cells. Measurements for which deviations between PXRD and DSC analysis was observed are indicated by bold numbers.

**Fig. 9:**
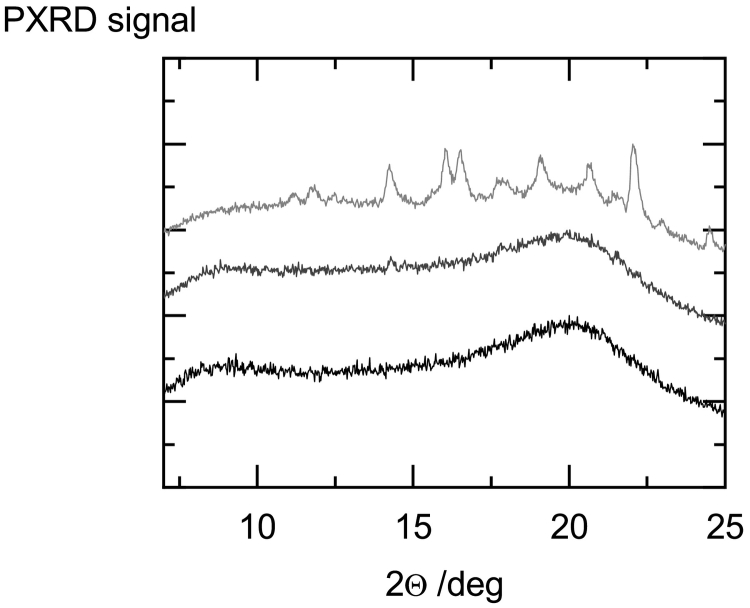
X-Ray diffractograms of HPC-UL/fenofibrate ASDs containing 5 wt% fenofibrate (black), 15 wt% fenofibrate (gray) and 30 wt% fenofibrate (light gray) after 12 weeks of storage.

Three example PXRD diffractograms of HPC-UL/fenofibrate ASDs obtained after 12 weeks of storage are shown in Fig. 9 illustrating the influence of the fenofibrate load on the degree of final crystallinity.

The fenofibrate/HPC-UL ASD with 5 wt% fenofibrate remained completely X-ray amorphous, the fenofibrate/HPC-UL ASD with 15 wt% fenofibrate was slightly crystalline and the fenofibrate/HPC-UL ASD with 30 wt% fenofibrate clearly crystallized.

The crystallinities of all ASDs determined via DSC function of time are shown in Table 6. The fenofibrate/HPC ASDs with 30 wt% fenofibrate load were already highly crystalline after the first week. This might be explained by the occurrence of amorphous phase separation (compare Fig. 5): The evolving fenofibrate-rich phase is almost pure according to the prediction, thus has a high molecular mobility (T_g,__f__enofibrate_ = −18 °C) and after demixing crystallizes as fast as the pure amorphous fenofibrate.

For the 5 wt% fenofibrate-loaded ASDs, only the polymers HPC-UL and HPC-SL successfully prevented the fenofibrate crystallization, whereas the other ASDs crystallized during storage.

**Fig. 10:**
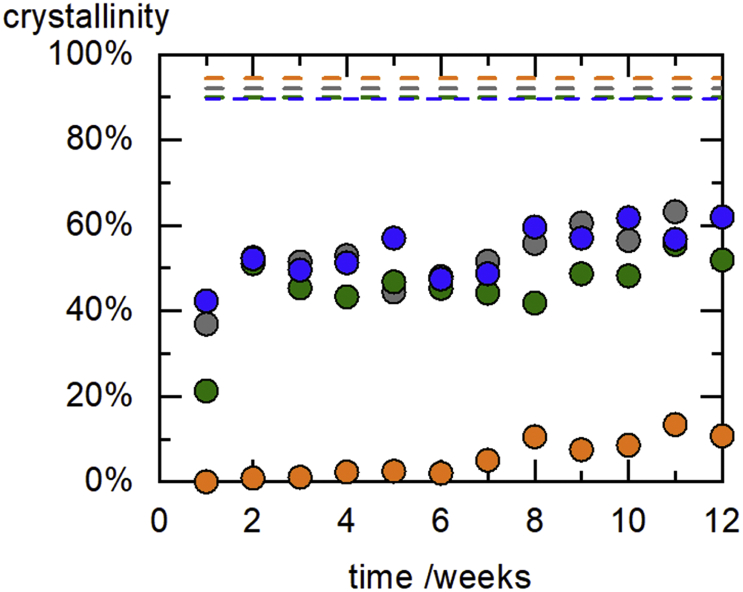
Time-dependent fenofibrate crystallinity in an HPC ASDs with 15 wt% fenofibrate. Orange (HPC-UL), blue (HPC-SSL), green (HPC-SL) and gray (HPC-L). (For interpretation of the references to colour in this figure legend, the reader is referred to the web version of this article.)

Fig. 10 shows the fenofibrate crystallinity in the e/HPC ASDs with 15 wt% fenofibrate (data from Table 6). HPC-UL ASDs with 15 wt% fenofibrate content crystallized significantly slower than the other ASDs (see Fig. 10). The crystallization velocity of fenofibrate in HPC-UL is much slower that in the other three HPC grades. Thus, HPC-UL seems to stabilize fenofibrate ASDs best. The kinetics of crystallization we observed in ASDs with HPC-SSL, HPC-SL and HPC-L does not follow a sigmoidal increase with time as known from other works (Luebbert and Sadowski, 2018; Yang et al., 2010), but jumps abruptly to values of 40% crystallinity within the first week of storage (Fig. 10) and then stays more or less constant over time. Only in the HPC-UL samples, the crystallinity only very slowly increases to maximal 10.7%. Due to a certain solubility of fenofibrate in the polymers, not all fenofibrate molecules will crystallize. That is why the maximum crystallinity is limited to values below 100% (predicted equilibrium crystallinities can be found in Table 6).

The sometimes-higher crystallinities observed in the DSC measurements compared to the X-ray measurements are attributed to recrystallization during heating, making an accurate quantification of crystals impossible (compare Fig. 8b).

Even the highest crystallinities observed in this work remained significantly below the predicted equilibrium crystallinities. This supports the hypothesis that HPC cannot suppress crystal nucleation but stabilizes an amorphous API very well against crystal growth even in presence of seed crystals. Very similar observations were also made during dissolution tests by Sarode et al. (Sarode et al., 2014b).

A reason for this might lie in the HPC-molecular-weight dependence of the amorphous solubility of fenofibrate (Fig. 7): Amorphous phase separation occurs for HPC grades with higher molecular weight, thus those ASDs are more likely to crystallize fast. In the low-M_w_ HPC-UL grade, smaller polymer chains might increase the amorphous solubility and thus prevent amorphous phase separation.

Table 6 shows that also itraconazole ASDs are best stabilized by HPC grades of lower- molecular weight: itraconazole ASDs with HPC-UL ASDs showed at maximum 2.7% crystallinity, the other grades up to 11.7% crystallinity. All itraconazole-containing samples remained X-ray amorphous throughout the entire 12 weeks of storage, thus all HPC grades could stabilize the investigated itraconazole ASDs for the investigated time period.

The API solubility in HPC is very low in both cases (Fig. 5 and Fig. 6), thus all ASDs from Table 6 were not thermodynamically stable and will eventually crystallize during storage. However, the glass transitions strongly differ between fenofibrate and itraconazole and thus also the ones of the respective ASDs. This kinetic stabilization is likely to be the reason for the enhanced physical stability of itraconazole/HPC-ASDs compared to fenofibrate/HPC-ASDs.

## 4 Conclusions

The hardly-detectable glass-transition temperatures of the pure HPC grades HPC-UL, HPC-SSL, HPC-SL and HPC-L were successfully determined for the first time by extrapolating the glass-transition temperatures of spray-dried HPC/PVPVA blends of different compositions and extrapolation to pure HPC (e.g. T_g,HPC-UL_ = 81.6 °C).

API/HPC phase diagrams were determined via DSC measurements and based on this, the thermodynamic model PC-SAFT was applied to model the crystalline API solubility, the glass-transition temperatures and to predict the regions of amorphous phase separation.

It was found that the API solubility at room temperature (25 °C) is 0.8 wt% in case of fenofibrate/HPC-UL and 0.0017 wt% in case of itraconazole/HPC-UL and thus, ASDs in the practically entire composition range were expected to crystallize after infinite time. Higher API concentrations lead to amorphous phase separation. This phenomenon was indirectly observed in the DSC measurements as well.

Long-term studies with spray-dried ASDs containing 5 wt%, 15 wt%, and 30 wt% were performed to validate the PC-SAFT predictions regarding API crystallization at storage conditions. As expected from the phase diagrams, most ASDs with fenofibrate crystallized during storage, while the itraconazole/HPC ASDs remained stable during three months of storage (due to kinetic stabilization and storage below the glass transition). Fenofibrate/HPC-UL ASDs with 15 wt% were significantly better stabilized against crystallization compared to the other investigated fenofibrate/HPC ASDs, although the crystalline fenofibrate solubilities in the different HPC grades are almost the same. The ASD with grade HPC-UL showed an enhanced stability compared to the one with the other HPC grades and is thus considered to be best suitable for generating ASD formulations with HPC grades. However, due to the overall low crystalline API solubilities, HPC appears more suitable as co-excipient to amorphous solid dispersions with e.g. PVPVA64 or HPMCAS.

Despite the long-term storage stability, further considerations on the in-vitro dissolution behavior (e.g. in biorelevant media) are required to ultimately evaluate the potential of HPC as excipient in ASD development.

## Declaration of Competing Interest

The authors declare that they have no known competing financial interests or personal relationships that could have appeared to influence the work reported in this paper.

## Appendix A Supplementary data

Supplementary material Image 1
