# Supplementary material for: Phase behavior of ASDs based on hydroxypropyl cellulose
Source: Int J Pharm X. 2020 Dec 19;3:100070. doi: 10.1016/j.ijpx.2020.100070 (PMC7773875; doi:10.1016/j.ijpx.2020.100070)
Supplement: Supplementary file 1 — Supplementary material [file mmc1.docx]

# *Supporting Information*

**Phase behavior of hydroxypropyl cellulose based ASDs**

**Christian Luebbert^1^, Edmont Stoyanov², Gabriele Sadowski^1,^³**

*^1^amofor GmbH, Otto-Hahn-Str. 15, D-44227 Dortmund, Germany, +49 231 9742 6365*

*^2^Nisso Chemical Europe GmbH, Berliner Allee 42, D-40212 Düsseldorf, Germany,* [*stoyanov@nisso-chem.de*](mailto:stoyanov@nisso-chem.de)*, +49 211 1306 9473*

*^3^TU Dortmund University, Laboratory of Thermodynamics, Emil-Figge-Str. 70, D-44227 Dortmund, Germany,* [*gabriele.sadowski@tu-dortmund.de*](mailto:gabriele.sadowski@tu-dortmund.de)*, +49 231 755 2635*

# DSC thermograms of pure HPC-UL

The polymers HPC-UL, HPC-L, HPC-SL and HPC-SSL were investigated via modulated differential scanning calorimetry (DSC) in a temperature range of 0 °C to 200 °C. Polymers were investigated in a heat (Ramp 1)  - cool (Ramp 2) - heat (Ramp 3) - procedure, the heating ramp was 2 °C/min with an underlying sinusoidal oscillation (1 minute oscillation period, amplitude 0.318 °C/min). A reversing and non-reversion heat flow signal was obtained from each measurement. The glass transition temperature was evaluated from the third heating ramp as shown in Figure S1 using the software TA Universal Analysis by TA Instruments.

Figure S1: DSC heat flow (green) and reversing heat flow (blue) of the first and third heating ramp of HPC-UL (2 K/min). The black lines mark the tangent construction for determining the glass transition using the software TA Universal Analysis.

The result of the DSC measurement of pure HPC-Ul is shown in Figure S1. In the first heating ramp, a broad endothermic event was observed between 25 °C and 100 °C in the heat flow (Figure S1), this endotherm is the evaporation of residual water from the sample.

After a complete evaporation of residual water and the extinction of thermal history at 200°C, the sample was cooled again and heated once more (Ramp 3). In this ramp. we observed no clear glass transition step, only a very slight kink in the slipe of the baseline. The software-based construction of baseline tangents yielded a T_g_ of 90.39°C with a T_g_ onset temperature of 80.60°C and a T_g_ offset temperature of 108.09°C. The in Figure S1 observed temperature window of the glass-transition (80.60°C < 108.09 °C) is very broad, it corresponds well to the value determined with the extrapolation of HPC-UL/PVPVA64 blends (T_g_ = 81.6 °C).

# Sorption equilibria of HPC-UL and different organic solvents

The equilibrium sorption values used for fitting the PC-SAFT parameters of HPC are summarized in Table S1. Values were determined via DVS as function of relative saturation (RS) at a temperature of 25 °C.

Table S1: Equilibrium sorption values of the investigated solvents in HPC-UL at 25 °C.

| **RS** | **w_water_** | **RS** | **w_ethanol_** | **RS** | **w_acetone_** | **RS** | **w_cyclohexane_** |
| --- | --- | --- | --- | --- | --- | --- | --- |
| 18.9% | 1.84% | 15.6% | 2.13% | 21.7% | 1.90% |  |  |
| 39.1% | 3.75% | 30.6% | 4.54% | 39.0% | 3.85% | 34.6% | 0.37% |
| 58.4% | 6.99% | 45.5% | 7.93% | 53.0% | 6.29% | 49.7% | 0.40% |
| 68.0% | 9.62% | 60.3% | 12.78% | 64.6% | 9.00% | 63.8% | 0.49% |
|  |  | 75.8% | 19.72% | 74.8% | 12.16% | 77.0% | 0.61% |

# DSC characterization of fenofibrate/HPC and itraconazole/HPC ASDs

The phase behaviour of fenofibrate/HPC-UL and itraconazole/HPC-UL ASDs was determined via DSC. Formulations with different API mass fractions (0.2, 0.4, 0.6, and 0.7) were prepared via ball milling. In total 100 mg of each ASD (API and HPC-UL) were weighed with an analytical balance in the desired ratio and then ball milled for 20 minutes at a frequency of 50 Hz. For each DSC experiment, approximately 10 mg of the ball milled ASDs were transferred to DSC aluminium pans, the samples were pressed into the pan to obtain an optimal contact with the sample pans. Four samples of each ASD were analysed in a heat-cool-heat procedure within a temperature range of -40 °C to 100 °C in case of fenofibrate and from 20°C-180°C in case of itraconazole ASDs. The heating ramps were modulated, and the modulation amplitude was set to a value so that the ramp was a heating-only ramp at an oscillation period of 1 minute. One sample was analysed with a heating rate of 1 K/min, two samples were analysed with a heating rate of 2 K/min to check for the measurement deviations and the fourth sample was analysed with a heating rate of 5 K/min.

Table S2: DSC method to analyse the ASDs.

| **Sample** | **Heating ramp** | **Oscillation period** | **Amplitude** | **Temperature range** |
| --- | --- | --- | --- | --- |
| FEN / HPC-UL | 1 K/min | 1 min | 0.159 K/min | -40 °C to 100 °C |
| FEN / HPC-UL | 2 K/min | 1 min | 0.318 K/min | -40 °C to 100 °C |
| FEN / HPC-UL | 5 K/min | 1 min | 0.796 K/min | -40 °C to 110 °C |
| ITR / HPC-UL | 1 K/min | 1 min | 0.159 K/min | 20 °C to 180 °C |
| ITR / HPC-UL | 2 K/min | 1 min | 0.318 K/min | 20 °C to 180 °C |
| ITR / HPC-UL | 5 K/min | 1 min | 0.796 K/min | 20 °C to 180 °C |

The melting temperature was obtained by extrapolating the dissolution event offset temperatures measured at the different heating rates to a theoretical equilibrium heating ramp of 0 K/min. The offset temperatures of the dissolution events were considered as the solubility temperatures. The equilibrium solubility temperatures of all HPC-UL ASD are summarized in Table S3.

Table S3: Equilibrium solubility temperatures and glass transitions of the different fenofibrate/HPC-UL and itraconazole/HPC-UL ASDs.

|  | **Fenofibrate + HPC-UL** | | **Itraconazole + HPC-UL** | |
| --- | --- | --- | --- | --- |
| **w_API_** | **T^SL^ /°C** | **T_g_ /°C** | **T^SL^ /°C** | **T_g_ /°C** |
| 0.2 | 80.50 | 11.31 | 162.37 | 57.20 |
| 0.4 | 80.97 | -18.59 | 162.98 | 58.26 |
| 0.6 | 81.70 | -18.31 | 162.91 | 58.45 |
| 0.7 | 81.56 | -18.41 |  |  |
| 1.0 | 80.78 | -18.44 | 168.75 | 59.25 |

It can be seen that the solubility temperatures do not decrease in any of the investigated fenofibrate ASDs compared to the pure fenofibrate melting temperature (80.78 °C), it even slightly increased in the 0.6 and 0.7-ASDs. The glass transition temperatures were like the ones of pure fenofibrate (‑18.44 °C). These findings qualitatively reveal weak intermolecular interactions and are a hint for the occurrence of amorphous phase separation in all ASDs except for the w_fenofibrate_ = 0.2 - ASD.

The itraconazole/HPC-UL ASDs showed a constant solubility temperature of 162.9 °C, this value lies 6 °C below the melting temperature of pure itraconazole. The glass transitions in itraconazole/HPC-UL ASDs are again in the vicinity of the glass transition of pure itraconazole.

The DSC curve of an itraconazole/HPC-UL ASD is shown in Figure S2 (the offset melting temperature shown in this figure is above the value in Table since this table contains the extrapolated values to 0 K/min).

Figure S2: DSC heat flow (green) and reversing heat flow (blue) of the itraconazole/HPC-UL ASD w_itraconazole_ = 0.4 at a heating ramp of 2 K/min.

During the first heating ramp (marked as Cycle 1), the itraconazole/HPC-UL ASD showed a recrystallization event at a temperature of 80 °C. Amorphous itraconazole recrystallized upon heating. The itraconazole in the ASD began to melt at a temperature of approximately 125 °C. All itraconazole crystals were fully dissolved in the HPC-UL matrix at a temperature of 163.92 °C. After a fast quench-cooling step, the ASD was reheated again (Cycle 3). The ASD now exhibited a clear glass transition at 58.82 °C. The exact glass transition was evaluated from the reversing heat flow signal of Cycle 3. Itraconazole shows a unique behaviour in the amorphous state which is also described in literature: It is one of the very few APIs forming liquid crystals (DSC characteristics described in Kozyra, A.; Mugheirbi, N. A.; Paluch, K. J.; Garbacz, G.; Tajber, L.: Phase Diagrams of Polymer-Dispersed Liquid Crystal Systems of Itraconazole/Component Immiscibility Induced by Molecular Anisotropy. *Mol. Pharmaceutics* **2018** 15 (11) 5192–5206. DOI: 10.1021/acs.molpharmaceut.8b00724). Also, the ASDs with HPC-UL exhibited those liquid crystalline structures, they are characterized by the two small melting events at temperatures of 74.81 °C (LC_Sm-N_) and 90.10 °C (LC_N-I_). All investigated ASDs showed itraconazole liquid crystals in the DSC analysis. A second glass transition was not observed in the entire heating procedure.

The heating rate dependence of the DSC signals was studied for the itraconazole/HPC-UL ASD w_itraconazole_ = 0.6 (Figure S3).

Figure S3: Heating rate dependence of the DSC thermograms itraconazole/HPC-UL w_itraconazole_ = 0.6 at 1 K/min (green), 2 K/min (purple and blue) and 5 K/min (black).

The solubility temperature decreased slightly from the melting temperature 169.75 °C (pure itraconazole) to 162.37°C in the 20wt.%-ASD. The melting temperatures decrease stronger compared to the solubility temperatures of the fenofibrate ASDs. The glass-transition temperature of the ASDs was slightly below the glass transition of pure itraconazole (59.25 °C). Like in the fenofibrate/HPC ASDs, HPC-UL again did not affect the glass transition temperature of the ASDs. We observed in all ASDs a glass transition close to that of pure itraconazole.
